# Antidiabetic Activity and In Silico Molecular Docking of Polyphenols from *Ammannia baccifera* L. subsp. Aegyptiaca (Willd.) Koehne Waste: Structure Elucidation of Undescribed Acylated Flavonol Diglucoside

**DOI:** 10.3390/plants11030452

**Published:** 2022-02-06

**Authors:** Noha Swilam, Mahmoud A. M. Nawwar, Rasha A. Radwan, Eman S. Mostafa

**Affiliations:** 1 Department of Pharmacognosy, Faculty of Pharmacy, The British University in Egypt (BUE), El Sherouk City 11837, Egypt; 2 National Research Centre, Department of Phytochemistry and Plant Systematic, Dokki 12622, Egypt; ma.el-moien@nrc.sci.eg; 3 Department of Biochemistry, Faculty of Pharmacy, Sinai University-Kantara Branch, El Ismailia 41611, Egypt; rashaaradwan@hotmail.com; 4 Department of Pharmacognosy, Faculty of Pharmacy, October University of Modern Sciences and Arts (MSA University), Giza 11787, Egypt; emostafa@msa.eun.eg

**Keywords:** *Ammania aegyptiaca*, myricetin 3-*O*-*β*-*^4^C_1_*-(6″-*O*-galloylglucopyranoside) 7-*O*-*β*-*^4^C_1_*-glucopyranoside, digestive enzymes, molecular docking, diabetes

## Abstract

Chemical investigation of the aerial parts of *Ammania aegyptiaca* ethanol extract (AEEE) showed high concentrations of polyphenol and flavonoid content, with notable antioxidant activity. Undescribed acylated diglucoside flavonol myricetin 3-*O*-*β*-*^4^C_1_*-(6″-*O*-galloyl glucopyranoside) 7-*O*-*β*-*^4^C_1_*-glucopyranoside (MGGG) was isolated from the aerial parts of AEEE, along with four known polyphenols that had not been characterized previously from AEEE. The inhibitory effects of MGGG, AEEE, and all compounds against *α*-amylase, pancreatic lipase and *β*-glucosidase were assessed. In addition, molecular docking was used to determine the inhibition of digestive enzymes, and this confirmed that the MGGG interacted strongly with the active site residues of these enzymes, with the highest binding free energy against *α*-amylase (−8.99 kcal/mol), as compared to the commercial drug acarbose (−5.04 kcal/mol), thus justifying its use in the potential management of diabetes. In streptozotocin (STZ)-induced diabetic rats, AEEE significantly decreased high serum glucose, *α*-amylase activity and serum liver and kidney function markers, as well as increasing insulin blood level. Moreover, AEEE improved the lipid profile of diabetic animals, increased superoxide dismutase (SOD) activity, and inhibited lipid peroxidation. Histopathological studies proved the decrease in pancreas damage and supported the biochemical findings. These results provide evidence that AEEE and MGGG possess potent antidiabetic activity, which warrants additional investigation.

## 1. Introduction

Diabetes mellitus (DM) is a major metabolic disorder leading to high morbidity and mortality rates around the world [1]. The incidence of diabetes in developing countries has reached epidemic proportions, and the International Diabetes Federation (IDF) expects an increase from 382 million people with diabetes to 592 million between 2013 and 2035 [2]. Diabetes is characterized by elevated plasma glucose concentrations, which are caused by insufficient insulin, insulin resistance, or both. In addition, abnormalities in various metabolic pathways [3]. As the disease progresses, it results in complications such as retinopathy, neuropathy, nephropathy, stroke, ischemic heart disease, peripheral vascular disease, and a variety of heterogeneous diseases [4], more than 95% of which are type 2 diabetes (T2D). It develops as a result of insulin resistance and pancreatic *β*-cell dysfunction, resulting in hyperglycemia [5]. Interestingly, there is a direct link between T2D and obesity. Obesity leads to increased cytokine production, fat deposition in body tissues and mitochondrial dysfunction, which results in insulin resistance and pancreatic *β*-cell apoptosis [6].

The current treatment for T2D is the usage of oral hypoglycemic drugs. Insulin replacement therapy is the main treatment for patients with type 1 diabetes [7]. However, the undesirable effects of such drugs create the need for alternative therapies with no side effects [8].

As a consequence, there is a growing interest in phytomedicine; plant extracts can be safer, easily available and affordable and have less incidence of adverse effects in comparison to synthetic antihyperglycemic drugs [9].

One beneficial approach to the management of diabetes is to reduce post-prandial hyperglycemia and prevent lipid digestion and absorption. This is clearly associated with the preclusion of obesity and obesity-related diseases [10]. Exploring the natural inhibitors of hydrolyzing enzymes in carbohydrate and lipid digestion may provide an attractive combinatorial therapeutic strategy for the management/prevention of post-prandial hyperglycemia and obesity. Moreover, DM appears to be an oxidative stress-related endocrine disorder, and using antioxidants may be beneficial in its prophylaxis and treatment [11]. The aforementioned studies reported that, in T2D, oxidative stress is increased due to chronic hyperglycemia, leading to the manufacturing of reactive oxygen species (ROS) in *β*-cells, thereby decreasing antioxidant activity via the enhancement of free radical generation [12]. Consequently, medicinal plants rich in antioxidant phytoconstituents can shield *β*-cells from reactive oxygen species (ROS) and avoid diabetes induced by ROS [13].

Increasing evidence suggests that plant polyphenols may have therapeutic antioxidant activities, which are related to their capacity to scavenge a wide range of ROS [14]. Thus, a relevant source of plant phenolics, as potential therapeutic agents that can be used to decrease blood glucose levels and lipids for the dual control of diabetes and obesity via digestive enzyme inhibition, specifically pancreatic *α*-amylase, intestinal *β*-glucosidase and pancreatic lipase inhibition [15], along with its ability to boost the body’s antioxidant system, has become an absolute necessity.

One of the famous plant genera that include phenolic-rich species is the genus *Ammannia* (Lythraceae). This genus is known to include species capable of synthesizing and accumulating a high percent of phenolics [16]. *Ammannia baccifera* L. subsp. aegyptiaca (Willd.) Koehne Waste (AE) is an annual herb distributed in tropical regions. In Egypt, it has been recorded in the Nile and Oasis geographical regions. It grows as a summer weed in rice fields, ditches and swamps [17]. This term is often used as a synonym for a variety of subspecies of *Ammannia baccifera* L., e.g., [18]. However, AE is an accepted species separated from the other three species known from Egypt by its sessile or subsessile flowers in compact axillary cymes. The type specimen of this species was collected from Damietta, Egypt [17]. In the available current literature, the phenolic profile and biological activities of AE have never been investigated. However, other species have been reported to have antidiabetic activity [19].

This study aimed to subject the flowering whole plant ethanol extract (AEEE) of AE to an intensive phytochemical investigation of its phenolic constituents and investigate the in vitro antioxidant activity of AEEE and the isolated constituents, along with an evaluation of their inhibitory effects and antidiabetic activity against the extra-pancreatic digestive enzymes and, thereby, potential antidiabetic activity. Molecular docking analysis was used to demarcate the activity of the tested compounds against digestive enzymes. Previous studies reported that phenolic compounds and flavonoids were able to bind to the pockets of *α*-amylase, *β*-glucosidase and pancreatic lipase, forming enzyme-inhibitors complexes [20,21,22]. Furthermore, the researchers evaluated the potential antidiabetic activity of the flowering whole plant AEEE in diabetic rats in order to assess its antidiabetic effects. This provides related data for further study regarding its utilization as an adjuvant therapy in the management of T2D. As the plant is a weed that mainly threatens water channels and rice fields, this study has focused on the exploitation of this plant waste.

## 2. Results

### 2.1. Total Phenolic and Flavonoid Contents

The phenolic content of AEEE was determined to be 380.5 ± 3.88 mg GAE/g, where R^2^ = 0.9985, and the equation for standard curve was Y = 0.0242x + 0.0211. In addition, the total flavonoid content in the AEEE was evaluated as 190 ± 2.31 mg CE/g, where R^2^ = 0.9989, and the equation for the standard curve was Y= 0.0048x + 0.0091. Thus, AEEE was found to be a source of a wide range of potent polyphenolic constituents.

### 2.2. Identification of Polyphenols from AEEE

**Figure 1:**
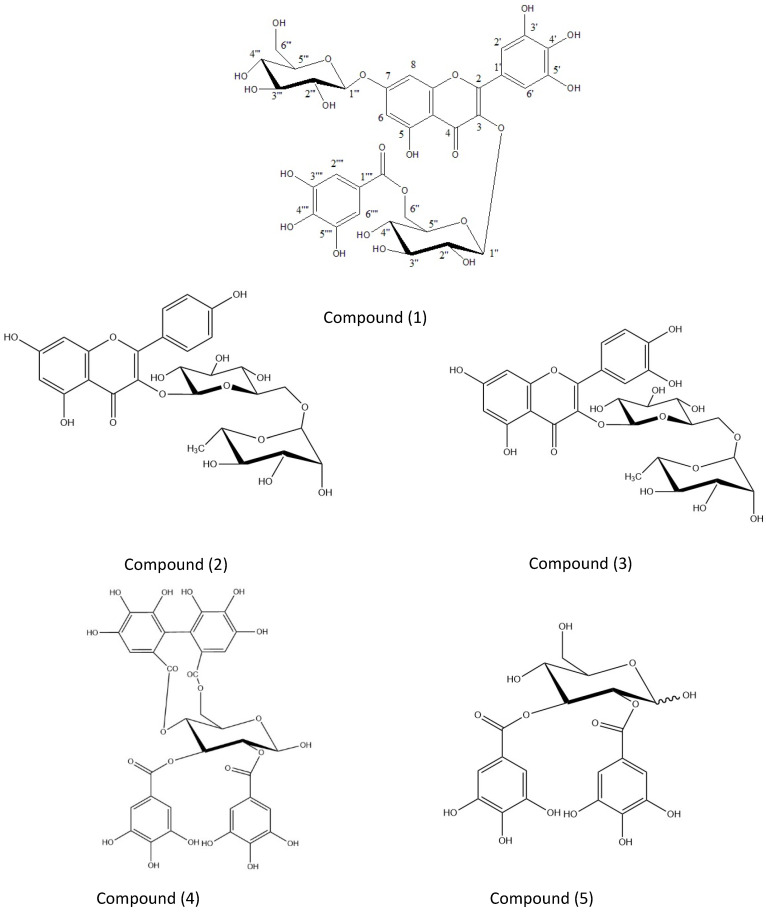
Myricetin3-*O*-*β*-*^4^C_1_*-(6′′-*O*-galloylglucopyranoside)-7-*O*-*β*-*^4^C_1-_*glucopyranoside (**1**), kaempfeol 3-*O*-rutinoside (**2**), quercetin 3-*O*-rutinoside (**3**), tellimagranidine-I (**4**) and 2,3-*α*, *β*-digalloy glucose (**5**).

A concentrated aqueous ethanol extract (3:1) of AEEE was prepared from fresh aerial samples of AE. The AEEE was fractionated over column chromatography on a Sephadex LH 20 using water/methanol mixtures of decreasing polarities. This was followed by the column fractionation of the 30 and 50% aqueous methanol Sephadex LH 20 column fractions over MCI gel, which afforded pure samples of compounds (**1**–**5**). The known compounds (**2**–**5**) showed chromatographic, UV absorption and hydrolytic data identical with those reported for kaempfeol 3-*O*-rutinoside **2** [23], quercetin 3-*O*-rutinoside **3** [24], tellimagranidine-I **4** [16] and 2,3-*α*,*β*-digalloy glucose **5** [25], respectively (Figure 1).

Compound **1**, a yellow amorphous powder, showed chromatographic properties (dark purple spot on paper chromatogram under UV light, turning reddish-orange when fumed with ammonia vapor and moderate migration in aqueous and organic solvents). The UV spectral analysis of **1** in methanol and upon the addition of shift reagents [16] confirmed the presence of a flavonol moiety, with a free hydroxyl at the C-4′ position (stable NaOMe spectrum) and a substituted hydroxyl at the C-7 position (showed no shift in UV with NaOAc). UV: λ_max_ (MeOH), (nm): 256, 356; +NaOAc: 257, 358; +NaOAc+H_3_BO_3_: 268, 390; +AlCl_3_: 269, 395; +AlCl_3_ +HCl: 271, 303, 401; NaOMe: 253, 404 nm. The acid hydrolysis of **1** yielded glucose (co-chromatography), myricetin and gallic acid (co-chromatography and ^1^H NMR). A negative ESI/MS for **1** was consistent with this view and showed a molecular ion peak at [M–H]^−^: 793.27, consistent with a molecular mass of 794. Structure **1** and the site of attachment of the galloyl moiety to the 3-*O*-glucoside moiety were confirmed by NMR analysis.^1^H NMR spectrum of **1** (DMSO-*d_6_*) showed two anomeric hexose proton signals at δ ppm and 5.35 and 5.02, each with (d, *J* = 8.5 Hz), corresponding to the anomeric glucoside moieties at the 3- and 7-positions of the flavonol moiety, respectively. In addition, the spectrum revealed a pair of methylene glucose proton signals at δ 4.26 (d, *J* = 12 Hz) and 4.12 (m), which were attributable to two 6′′- glucose carbons whose germinal hydroxyl groups are acylated by the galloyl moiety. The signals of the two methylene protons in the 7-*O*-glucoside moiety and the remainder of the glucose protons overlapped with the water protons’ signal and showed an up-field shift to the region from δ 3.3 to 3.9. The characteristic protons of the galloyl moiety displayed a singlet signal at δ 7.02. In addition, the 7-*O*-substituted myricetin moiety was evidenced by proton signals at δ 6.3 (d, *J* = 2.5 Hz) and 6.75 (d, *J* = 2.5 Hz), which can be attributed to the H-6 and H-8 of this moiety [16]. ^13^C NMR of **1** confirmed its structure. It exhibited twelve glucose carbon signals.

The two *β*-glucose anomers at positions C-7 and C-3 were detected from the downfield resonances at δ ppm 99.3 and 102.9, respectively, while the methylene C-6 ′′′ glucose carbon with a free hydroxyl and the acylated methylene glucose C-6 ′′ carbon were found resonating up-field, at δ ppm 60.2 and 63.2, respectively. Acylation by gallic acid resulted in the de-shielding of the second resonance. The remaining glucose carbons were found resonating at a δ ppm value close to that of the flavonol 3,7-di-*O*-glycoside [16]. The presence of only one galloyl moiety in **1** followed from the single carboxyl carbon resonance at δ 166.5 and from the recognized, characteristic pattern of the remaining galloyl carbons. Substitutions at the 3- and 7-positions of myricetin were evidenced by the relative up-field shifts of the C-3 and C-7 signals to δ 134.4 and 161.6, respectively. Down-field shifts in the signals were detected corresponding to the *o*- and *p*-carbons of the flavonol moiety in **1,** in comparison with the corresponding signals in free myricetin [16].

Furthermore, the *^4^C_1_* conformation of the two glucose moieties was confirmed by the measured chemical shift values of the carbon resonances. The above data confirm that the substitution pattern in **1** is typical of that present in quercetin 3-*O*-*β*-*^4^C_1_*-(6′′-*O*-galloyl glucopyranoside)-7-*O*-*β*-*^4^C_1_*-glucopyranoside, which was previously isolated from the same plant extract [16]. That the galloylated glucose linked to the hydroxyl group at C-3 of the aglycone was unambiguously confirmed by a 3 *J* long-range correlation between its anomeric proton (δ ppm 5.35) and C-3 (δ ppm 134.4) of the myricetin moiety in the hetronuclear multi bond connectivity (HMBC) spectrum. This spectrum also showed a cross peak correlating to the second anomeric proton resonance at δ 5.02 to the myricetin carbon C-7 at δ ppm 161.6. Consequently, **1** is identified as myricetin 3-*O*-*β*-*^4^C_1_*-(6′′-*O*-galloyl glucopyranoside)-7-*O*-*β*-*^4^C_1_*-glucopyranoside, which has not been reported previously in nature Figure 1.

### 2.3. Molecular Docking

**Figure 2:**
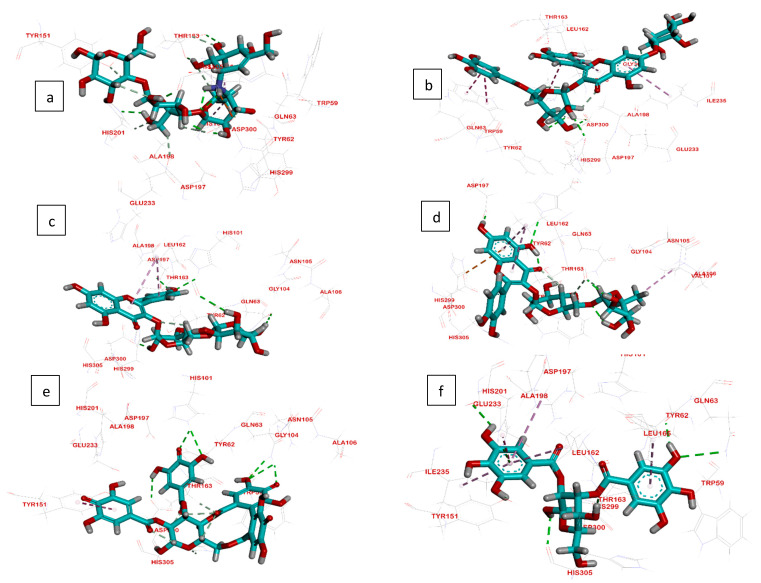
Molecular docking interactions of isolated compounds (**1**–**5**) with amino acid residues in the active site of α-amylase as 3D diagram. (**a**) Standard Acarbose (**b**) MGGG (**c**) Kaempfeol 3-*O*-rutinoside (**d**) Quercetin 3-*O*-rutinoside (**e**) Tellimagranidine-I (**f**) 2,3-*α*, *β*-digalloy glucose. The green dashed lines stand for hydrogen bonds and the purple dashed lines stand for pi interactions.

**Figure 3:**
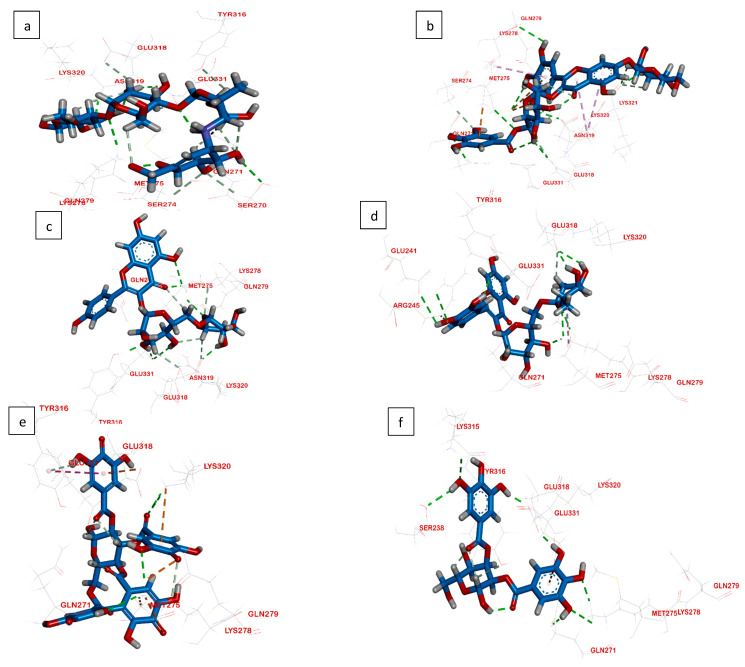
Molecular docking interactions of isolated compounds (**1**–**5**) with amino acid residues in the active site of *β*-glucosidase as 3D diagram. (**a**) Standard Acarbose (**b**) MGGG (**c**) Kaempfeol 3-*O*-rutinoside (**d**) Quercetin 3-*O*-rutinoside (**e**) Tellimagranidine-I (**f**) 2,3-*α*, *β*-digalloy glucose. The green dashed lines stand for hydrogen bonds and the purple dashed lines stand for pi interactions.

**Figure 4:**
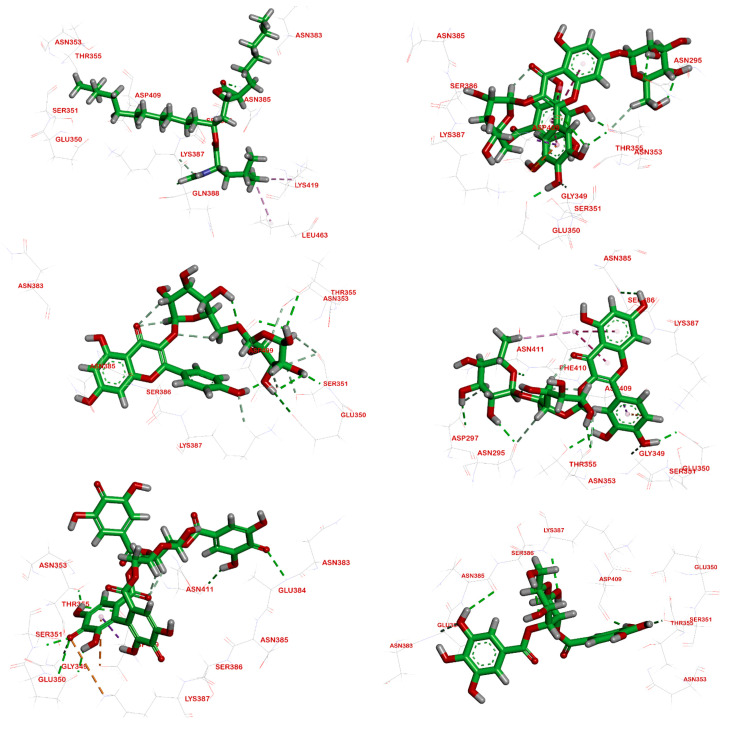
Molecular docking interactions of isolated compounds (**1**–**5**) with amino acid residues in the active site of pancreatic lipase as 3D diagram. (**a**) Standard Acarbose (**b**) MGGG (**c**) Kaempfeol 3-*O*-rutinoside (**d**) Quercetin 3-*O*-rutinoside (**e**) Tellimagranidine-I (**f**) 2,3-*α*, *β*-digalloy glucose. The green dashed lines stand for hydrogen bonds and the purple dashed lines stand for pi interactions.

Computational docking studies were utilized to predict the binding mode, affinity and binding free energy (∆G) of the tested compounds with pancreatic *α*-amylase, intestinal *β*-glucosidase and pancreatic lipase. All the molecular docking results regarding interactions between the isolated compounds (**1**–**5**), reference standards and three digestive enzymes’ binding sites, with some relevant parameters, are summarized in Table 1, Table 2 and Table 3 and Figure 2, Figure 3 and Figure 4.

#### 2.3.1. Interactions Analysis with α-Amylase

In accordance with the in vitro studies, the docking results indicated that compounds **(1–5)** bind to the enzyme active site with the lowest binding energies of −8.99, −7.07, −6.43, −6.47 and −6.50 kcal/mol, respectively (Figure 2). Compounds **1**, **4** and **5** were the first to be reported as inhibitory against *α*-amylase.

Among all isolates, MGGG exhibited the highest docking score (−8.99 kcal/mol), which is higher than that of the reference standard, acarbose (−8.13 kcal/mol). The binding of MGGG to α-amylase was greater than those of β-glucosidase and pancreatic lipase (−6.43 and −7.70 kcal/mol, respectively).

The active site of α-amylase consists mainly of Ala198, Asp197, Glu233, His201, His299, Tyr62, Asp300, Thr163, His101 and His305 [26]. As shown in Figure 2, all these residues participated in the binding of the enzyme to the docked compounds.

The hydroxyl groups at C-5 and C-6 of the valienamine moiety of the reference standard, acarbose, formed two hydrogen bonds with Thr163. In addition, the C-2 hydroxyl group in the O-4,6-dideoxy α-D-glucopyranosyl moiety interacted with Asp300 via a hydrogen bond. The methyl group in the O-4,6-dideoxy α-D-glucopyranosyl moiety interacted with Leu165 and His101 by forming Pi-alkyl interactions. Furthermore, the C-2, C-3 and C-6 hydroxyl groups in the O-α-D-glucopyranosyl moiety formed three hydrogen bonds with His305, Asp300 and His201 (Figure 2a).

In MGGG, the C-2, C-3 and C-4 sugar hydroxyl groups of the galloylated glucose moiety formed three hydrogen bonds with Asp300 and Asp197, while the chromone ring formed two Pi–alkyl interactions with Ile235 and Leu162. On the other hand, the galloyl moiety interacted with Trp59 via a Pi–Pi interaction, and the flavonol B-ring formed another Pi–Pi interaction with His305 (Figure 2b).

#### 2.3.2. Interactions Analysis with β-Glucosidase

Confirming the in vitro results, the docking scores for the isolated compounds **1**–**5** were −6.43, −5.28, −5.42, −5.36 and −5.70 kcal/mol, respectively (Figure 3). All isolates were first to be reported as inhibitory against *β*-glucosidase. Among all isolates, MGGG exhibited the highest docking score (−6.43 kcal/mol), which is higher than that of the reference standard, acarbose (−5.04 kcal/mol).

Met275, Lys320, Asn319, Tyr316, Ser274, Gln271, Ser270, Glu331 and Lys278 play a significant role in binding to the co-crystallized ligand on the active site of *β-g*lucosidase [27]. As shown in Figure 3, all these residues, except Ser270, participated in the binding of the enzyme to the inhibitor isolates.

The best pose on the part of the reference (acarbose) was found to form five hydrogen bonds through its hydroxyl groups. The hydroxyl groups attached to C-5 and C-6 of the valienamine moiety formed two hydrogen bonds with Gln271 and Ser270. Additionally, the C-2 hydroxyl group in the O-4,6-dideoxy α-D-glucopyranosyl moiety interacted with Glu331 via a hydrogen bond. The hydroxyl group attached to C-2 in the terminal α-D-glucose moiety formed a hydrogen bond with Asn319, while the terminal acetal linkage in acarbose interacted with Lys278 via one hydrogen bond (Figure 3a).

On the other hand, regarding the new isolate, the C-4 sugar hydroxyl group in the galloylated glucose moiety formed a hydrogen bond with Glu318, while the galloyl moiety interacted with Lys278 and Gly271 via two hydrogen bonds and with Lys287 via Pi–cation interaction. Additionally, B-ring hydroxyl groups formed four hydrogen bonds with Asn319, Met275, Gln279 and Lys278, as well as a Pi–sulfur interaction with Met275, While the C-3hydroxyl group in the 7-*O*-*β*-glucopyranoside moiety formed a hydrogen bond with Lys321, the chromone ring formed two Pi–alkyl interactions with Lys320 (Figure 3b). The docking results confirmed that MGGG had similarities with acarbose because they interacted with several key amino acids residues of *β*-glucosidase, Lys278, Asn319 and Gln271, suggesting that those three amino acid residues may play an important role in the inhibitory activities of these two compounds.

#### 2.3.3. Interactions Analysis with Pancreatic Lipase

The isolated compounds (**1**–**5**) were able to bind to the active site of pancreatic lipase. The binding free energy values were −7.70, −7.20, −7.39, −5.43 and −5.51 kcal/mol, respectively, confirming the in vitro data (Figure 4). All isolates were first to be reported as inhibitory against pancreatic lipase except compound (**3**) [21].

The key residues of pancreatic lipase’s active pocket are Asn383, Asn353, Glu350, Asn385, Asp409, Lys419, Leu463, Thr355, Ser 351, Gln388, and Lys 387 [28]. As shown in Figure 4, all these residues, except Gln388, Leu463 and Lys419, participated in the enzyme-inhibitor complexes.

**Figure 5:**
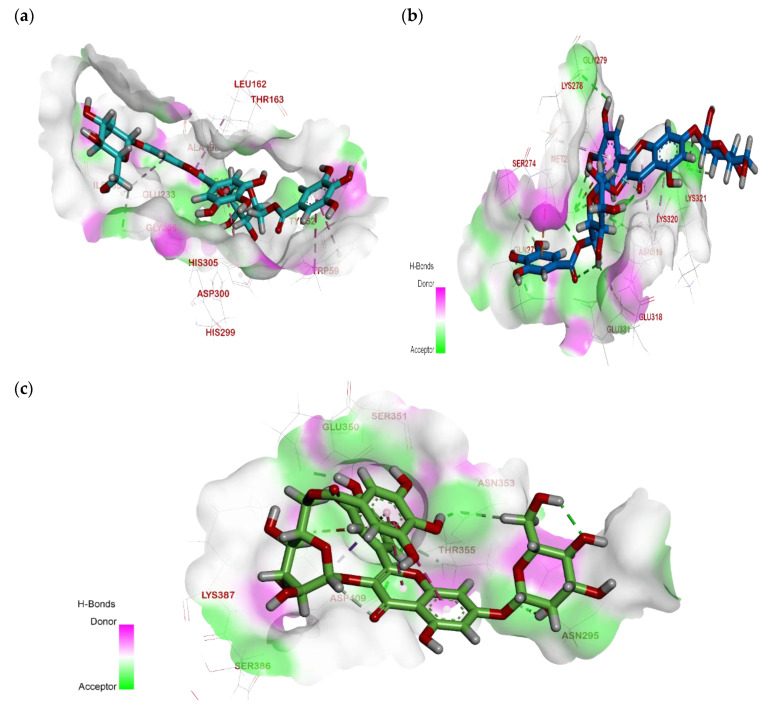
Mapping surface showing MGGG occupying the active pocket of *α*-amylase (**a**), *β*-glucosidase (**b**) and pancreatic lipase (**c**), respectively.

Among all the isolates, MGGG exhibited the highest docking score (−7.70 kcal/mol), which was higher than that of the reference standard, orlistat (−6.66 kcal/mol). The docking results for the chosen pose of the reference, orlistat, in the binding site of pancreatic lipase showed an interaction with a carbonyl group of the formamide moiety, forming one hydrogen bond with Gln388, while another carbonyl group formed a hydrogen bond with Asn385. The hydrophobic aliphatic tail showed a hydrophobic interaction with Leu463 and Lys419 (Figure 4a). The docking of MGGG into the binding site of pancreatic lipase revealed an extensive network of hydrogen bonds, in which the hydroxyl groups in the B-ring formed three hydrogen bonds with Glu350, Thr355 and Asp409, as well as a Pi–anion interaction with Asp409, while the hydroxyl group attached at C-2 in the 7-O-*β*-D-glucopyranose moiety formed a hydrogen bond with Asn295; additionally, the hydroxyl group in the galloyl moiety formed a hydrogen bond with Asn353 (Figure 4b). For all tested digestive enzymes, a surface mapping technique was carried out to show MGGG occupying the active pocket of these enzymes (Figure 5).

### 2.4. In Vitro Studies

#### 2.4.1. DPPH Assay

The antioxidant activity of AEEE, MGGG and the isolated compounds (**2**–**5**) were evaluated via DPPH assay. The IC_50_ values were 6.12 ± 0.83 µg/mL, 2.37 ± 0.56 µg/mL, >100 ± 13.67 µg/mL, 8.81 ± 1.05 µg/mL, 4.64 ± 0.39 µg/mL and 4.81 ± 0.73 µg/mL, respectively, as compared to Vitamin C (the positive control), with an IC_50_ of 1.83 ±1.41 μg/mL, reflecting the potent antioxidant activity of MGGG.

#### 2.4.2. ORAC Assay

The antioxidant activity of AEEE, MGGG, and the separated compounds (**2**–**5**) was evaluated using the more specialized ORAC assay. The IC_50_ values were 5.92 ± 1.03 µg/mL, 2.01 ± 0.23 µg/mL, >100 ± 10.17µg/mL, 8.02 ± 0.85 µg/mL, 4.14 ± 1.27 µg/mL and 4.43 ± 0.68 µg/mL respectively. All values were lower than that of Trolox (the positive control), which had an IC_50_ of 28.0 ± 14.31µg/mL, further endorsing the potent antioxidant activity of the new MGGG.

#### 2.4.3. Reducing Power Assay

**Figure 6:**
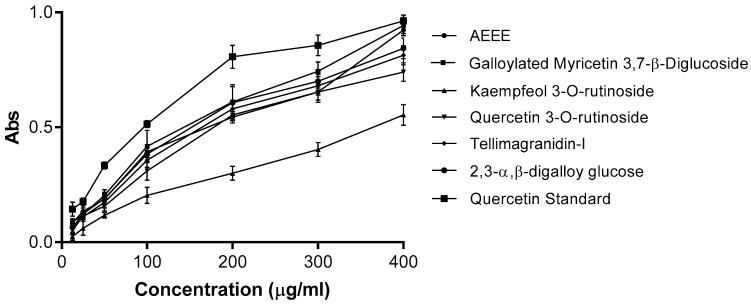
Reducing power of AEEE and isolated compounds (**1**–**5**) compared with quercetin as standard. Results are given as mean ± SD of three replicate analyses.

The reducing power of phenolic compounds suggests their antioxidant activity. In the reducing-power assay, the more antioxidant compounds convert the oxidated form of iron (Fe^+3^) in ferric chloride to ferrous (Fe^+2^), which leads to an increase in colored ferrous-TPTZ complexes. Figure 6 showed that AEEE, MGGG and the isolated compounds (**2**–**5**) had concentration-dependent reducing power. Additionally, MGGG showed the highest activity, with a reducing power comparable to that of standard quercetin.

#### 2.4.4. In Vitro Enzyme Assays

In the current study, AEEE and its isolated compounds exhibited anti-*α*-amylase, anti-*β*- glucosidase and anti-pancreatic lipase activities (Table 4).

### 2.5. In Vivo Assays

#### 2.5.1. Acute Oral Toxicity

The oral administration of AEEE showed no signs of toxicity or mortality, with no significant changes in neurological and behavioral responses in any tested group during the 48 h study at up to 2000 mg/kg body weight of the extract. Therefore, doses of 250 and 500 mg/kg were selected for subsequent antidiabetic studies.

#### 2.5.2. Effect of AEEE on Body Weight

**Figure 7:**
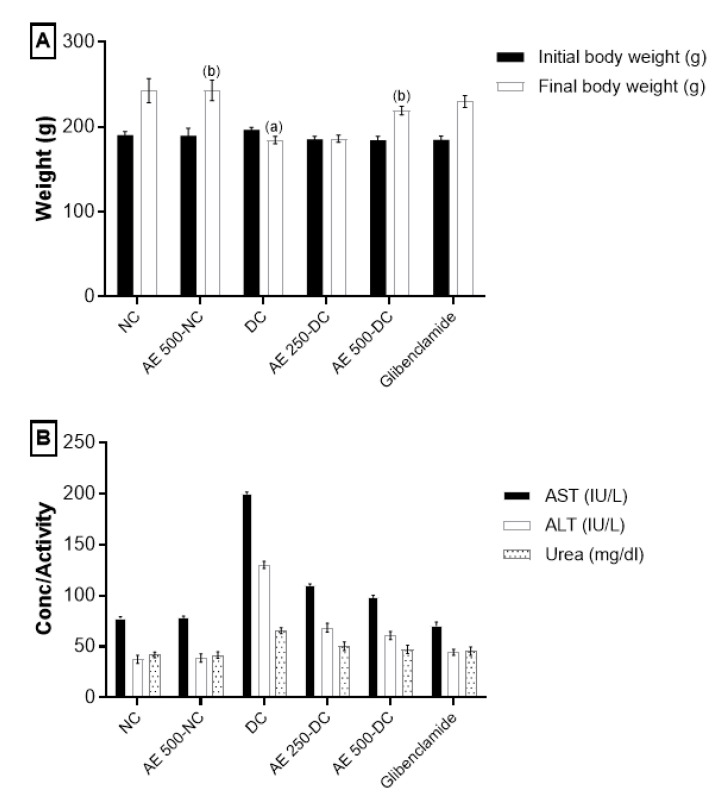
(**A**) Effect of AEEE on body weight during the experimental period (28 days). (**B**) Effect of AEEE on serum liver and kidney function markers. Results are stated as mean ± S.D. (*n* = 6). Results are considered significantly different at *p* < 0.05. (a) is statistically different from NC group; (b) is statistically different from DC group.

Treatment with AEEE at 500 mg/kg body weight significantly inhibited the reduction in the body weight induced by STZ, as compared to DC (Figure 7A).

#### 2.5.3. Effect of AEEE on Liver and Kidney Function Markers

No significant changes in the liver and kidney function tests were observed. After two weeks of treatment, diabetic groups treated with 250 or 500 mg/kg AEEE exhibited alleviated hepatocellular damage caused by STZ in the form of a reduction in AST and ALT levels [29]. In addition, these groups reversed their high creatinine levels from 1.3 to 0.76 ug/mL. Moreover, the administration of the extract preserved the values of serum AST, ALT, urea and creatinine, illustrating its non-toxic nature, as shown in Figure 7B. Additionally, AEEE showed insignificant changes in both liver and kidney function tests, with irrelevant alterations in all parameters addressed in the present investigation.

#### 2.5.4. Effect of AEEE on Serum Blood Glucose, Insulin and α-Amylase

**Figure 8:**
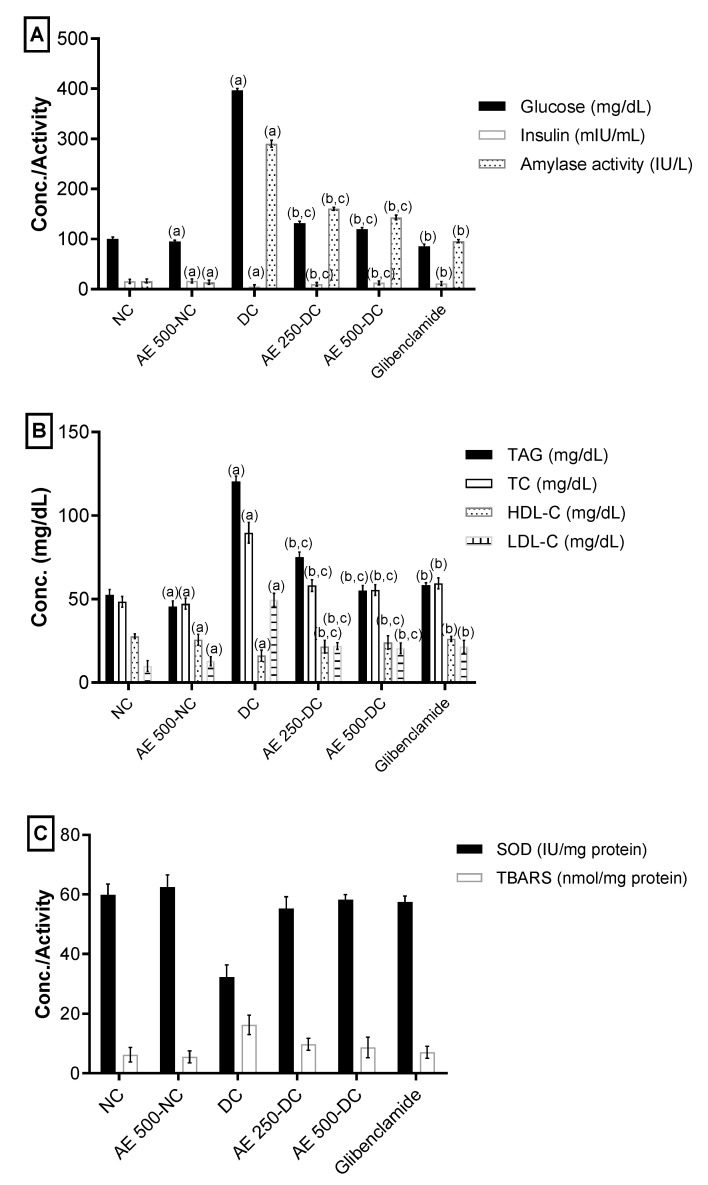
(**A**) Effect of AEEE on serum blood glucose, insulin and *α*-amylase. (**B**)Effect of AEEE on serum lipid profile. (**C**) Effect of AEEE on oxidative stress markers of pancreas. Results are stated as mean ± S.D, (*n* = 6). Results are considered significantly different at *p* < 0.05. (a) is statistically different from NC group; (b) is statistically different from DC group.

The serum glucose levels of STZ-induced diabetic rats in Groups IV and V remarkably decreased after the administration of 250 and 500 mg/kg of AEEE, with increased insulin levels and reduced *α*-amylase activity in comparison with the DC group. The percentage changes from DC were 66.8%, 91.6%, 44.7%, 69.8%, 158% and 50.7%, respectively. Nevertheless, there was a significant difference between the AEEE-treated diabetic groups and the glibenclamide group (VI) in all three measured parameters, as shown in Figure 8A.

#### 2.5.5. Effect of AEEE on Serum Lipid Profile

The TG, TC and LDL-C levels exhibited a notable reduction in diabetic groups treated with 250 and 500 mg/kg of AEEE, with an elevation in HDL-C levels relative to the DC group. The percentage changes from DC were as follows: 37.67, 34.93, 33.52, 55.58, 54.2, 38.04, 51.69 and 58.98, respectively. Furthermore, there was a significant difference between the AEEE-treated diabetic groups and the glibenclamide group (VI) in all lipid profile parameters, as shown in Figure 8B.

#### 2.5.6. Effect of AEEE on Oxidative Stress Markers of the Pancreas

A remarkable increase in antioxidant enzyme activity (SOD) and a significant reduction in TBARS manufacture in pancreatic tissues of the DC group versus NC group was observed (Figure 8). The AEEE-treated diabetic groups exhibited increased SOD activity and the significantly suppressed formation of TBARS in comparison with the DC group. The percentage changes from diabetic groups for the 250 and 500 mg/kg doses were 71.6, 42.78, 80.9, and 47.45, respectively. Furthermore, there was a significant difference between the AEEE-treated diabetic groups and the glibenclamide group (VI) in oxidative stress parameters (Figure 8C).

#### 2.5.7. Effect of AEEE on Pancreas Histopathological Examination

**Figure 9:**
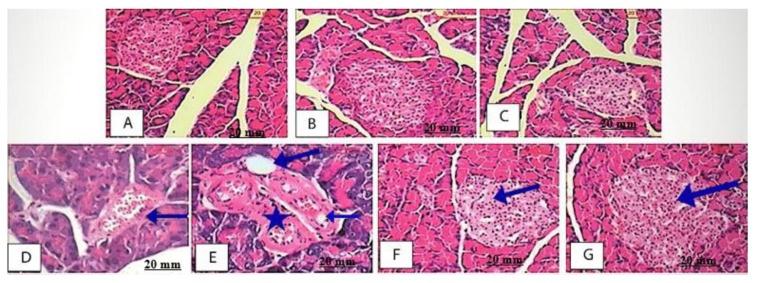
The histological investigation of pancreas. (**A**–**C**): Pancreas sections of NC, AE500-NC and AE 250-NC groups respectively, showing dense staining acinar cells and a light-staining islet of Langerhans; (**D**): Diabetic rat showing the acinar cells around the islets though seemed to be in normal proportion with up normal morphology with shrunken islet is and intra islet hemorrhage (blue arrow). (**E**): Diabetic rat showing degenerative islet of Langerhans (asterisk) with different vacuoles size (long arrow) and hemorrhage (short arrow); (**F**,**G**): AE 500-DC rat showing the exocrine pancreas appearing.

The pancreas histopathological examination of the NC, AE500-NC and AE250-NC groups showed a normal image (Figure 9A–C). The Islets of Langerhans appear as circular shapes with a normal cell lining, while the exocrine components and the interlobular duct surrounded with the supporting tissue appeared well organized and to have a normal morphology (Figure 9A,C). The image morphometry showed that the area of the mean islet in non-diabetic rats was (145.21 ± 3.49) mm^2^, whereas for AE500-NC and AE250-NC groups, these values were (175.67 ± 1.85) and (167.89 ± 1.34) mm^2^, respectively. On the other hand, a histopathological examination of the diabetic rats showed acinar cells around the islets with abnormal morphology. The cells of the islets were in a degenerative form, with asymmetrical vacuoles exhibiting intra-islet hemorrhage, reduced islet cell size and reduced *β*-cell number (Figure 9D,E). The diabetic rats’ mean islets area was (110.65 ± 9.41) mm^2^, which was smaller than that for normal rats. The pancreas sections of the AE500-DC and AE250-DC groups were microscopically investigated, suggesting the protection of the islets due to the recovery of their size, with *β*–cell repair (Figure 9F,G). This regeneration of the *β*-cells was more obvious at a higher dose, whereas with the mean islets areas for AE500-DC and AE250-DC groups were (185.33 ± 10.41) and (180.12 ± 6.52) mm^2^, respectively.

## 3. Discussion

Currently, the search for natural compounds that have both antidiabetic and antioxidant activities, along with fewer side effects as compared to traditional medications, remains challenging. Polyphenols, particularly flavonoids, are suggested as better therapeutic agents in the management of free radical-mediated diseases, particularly diabetes mellitus and its chronic complications, due to their potent antioxidant activity, which was demonstrated both in vitro and in animal model studies [30]. They are hydroxylated phenolic substances, and the hydroxyl group mediates their antioxidant effects by scavenging free radicals via chelating metal ions [31].

Flavonols, with MGGG the sub-class belonging to flavonoids, are effective as antioxidant and antidiabetic agents, mostly due to their chemical structure. Both their configuration and the total number of hydroxyl groups increased both of these activities and substantially regulated the mechanisms of radical scavenging [32] and antidiabetic effects. Thus, the total number of hydroxyl groups, hydroxyl configuration, the catechol structure in the B-ring, the C-2-C-3 double bond, and the C-4 ketonic functional group is essential in the bioactivity of flavonoids, especially regarding their antidiabetic effect [33]. Myricetin significantly improves insulin resistance, in addition to its antioxidant, anti-inflammatory and aldose reductase inhibitory actions [34].

As mentioned above, AE is an annual herb that grows as a summer weed in rice fields. Despite the reported antidiabetic activity of other *Ammannia* species, there are no data in the literature on AE’s phytoconstituents, antidiabetic activity or mechanism of action. In the current study, a phytochemical investigation of AEEE resulted in the isolation of a unique acylated flavonoid, myricetin 3-*O*-*β*-*^4^C_1_*-(6′′-*O*-galloyl glucopyranosid) 7-*O*-*β*-*^4^C_1_*-glucopyranoside, along with four additional known phenolics. A significant antioxidant potential of AEEE and polyphenols **2**–**5** was found, and the highest antioxidant potential was exhibited by MGGG, with IC5_0_ values of 2.37 ± 0.56, 2.01 ± 0.23 and 158.13 ± 2.82 µg/mL in comparison to a standard against DPPH, ORAC and ferrous reducing assay, respectively.

Several reports showed that the radical scavenging capacities increased with an increase in the number of phenolic hydroxyl groups; this was observed for the three classes of isolated compounds: flavonoids, gallotannins and ellagitannins [35]. Moreover, the antioxidant activity increased as the number of galloyl units increased; however, it was not affected by their position or the presence of hemiacetal hydroxyl and 4,6-*O*-HHDP groups. Additionally, the presence of two adjacent phenolic hydroxyl groups on the galloyl moiety was significant, and this was justified by the higher antioxidant capacity of tellimagrandin-I as compared to nilocitin and the potential of MGGG. Flavonoids with potent antioxidant activity were shown to be effective in the management of diabetes [36].

The docking results for the tested compounds (**1**–**5**) with the digestive enzymes provided solid information about the binding mode, which aligned correctly with the in vitro experimental results. For instance, the isolates showed a moderate to strong binding mode, with a docking score (∆G) range from −6.43 to −8.99 kcal/mol. It was observed that the tested candidates were well accommodated inside the active site of the digestive enzymes, displaying geometric complementarity and excellent mapping, which increased their inhibitory activity and was involved in various types of interactions with the active site residues of the target enzymes. In addition, among the tested enzymes, MGGG showed an RMSD value lower than 2.00 Å, which confirmed its occupancy in the original site of the crystal ligand.

From the point of view of the molecular structure of MGGG, the presence of multiple polar hydroxyl groups is very important for the stabilization of candidates inside the targeted sites of the tested digestive enzymes due to the availability of the electron-donating groups (-OH) forming an electronegative and electron cloud system inside the pocket, which can engage in fitted stabilized interactions with polar and charged amino acids. This justifies the higher inhibitory potential of MGGG as compared to the standard inhibitor used for digestive enzymes. In addition, the docking results revealed that the hydroxyl groups of the B-ring, galloyl glucose and *β*-D-glucopyranose moieties play a central role in its binding to the digestive enzymes by forming additional hydrogen bonds and Pi interactions that can fit the new isolate into the targeted pocket, consisting of the common amino acids between the reference standard and MGGG in *α*-amylase (Asp300 and His305) and *β*-glucosidase (Lys278, Gln271 and Asn319).

In terms of binding energy and affinity, as compared with the standards orlistat and acarbose, MGGG showed higher binding free energies for all tested digestive enzymes, with the highest docking score against *α*-amylase (∆G= −8.99 kcal mol) and the lowest docking score against *β*-glucosidase (∆G= −6.43 kcal mol). While the other candidates show moderate docking scores in all targets, compound **4** shows weak binding due to its structural bulkiness, which prevents perfect fitting inside the targeted pocket.

Consequently, the in silico study showed a promising result for the antidiabetic and anti-obesity drug discovery process. In fact, MGGG may prove to be a potential candidate for decreasing the activity of digestive enzymes.

The in vivo study showed that glucose levels were significantly reduced and insulin levels were elevated after the administration of AEEE as compared to the DC group. The histopathological results further confirmed this, demonstrating that the structural integrity of the islets of Langerhans was recovered. Furthermore, AEE decreased *α*-amylase activity. Phenolics were reported to decrease the activity of digestive enzymes [37,38], and this is in full agreement with the in vitro assay and molecular docking results.

Pancreatic amylase and intestinal glucosidase are crucial enzymes involved in glucose formation [39]. *β*-glucosidases catalyze the breakdown of alkyl and aryl-*β*-glycosides, disaccharides and short-chain oligosaccharides via the dual activities of hydrolysis and transglycosylation [40]. Therefore, the study of *β*-glucosidase inhibitors is important for the treatment of type 2 diabetes [41]. However, the effect of inhibitors on *β*-glucosidase has been scantly illustrated. The phenolic and flavonoid content in roselle was responsible for the inhibitory activity *α*-/*β*-glucosidase.

Pancreatic *α*-amylase catalyzes the first step in the starch breakdown. The suppression of intestinal *α*-amylase activity hinders starches’ and oligosaccharides’ breakdown into monosaccharides before absorption. This results in the profound control of T2D. Acarbose, an oral hypoglycemic agent, is used for the inhibition of *α*-amylase [42]. Flavonoids such as myricetin, the core of the new isolate MGGG; luteolin and quercetin were potent inhibitors. In fact, pancreatic lipase plays a key role in triglyceride absorption in the small intestine [43]. Thereby, the hindrance of triglyceride absorption via lipase inhibition is a major approach to avoiding obesity and the management of T2D. The commercial drug orlistat strongly inhibits the activity of pancreatic lipase [44]. Pancreatic lipase-inhibitory activity has been attributed to various types of phytochemicals, such as saponins, polyphenols and terpenes [45].

The in vitro enzymatic inhibition results showed that AEEE and all isolates **1**–**5** possessed antidiabetic and potential anti-obesity activities based on the inhibition of pancreatic lipase, *β*-glucosidase and *α*-amylase. Except for compound **3** [46], AEEE and the isolates (**1**, **4**, **5**) were first reported on the three enzymes. Moreover, a significant difference existed between AEEE and its isolates, as shown in (Table 2). Acylated flavonoids, the class of the new isolate MGGG and its core (myricetin) were previously reported to have strong antidiabetic activity [34,47]. In fact, MGGG exhibited the highest percentage of inhibition in all three enzyme assays, with increased antidiabetic and potential anti-obesity properties as compared to the reference standards acarbose and orlistat (Table 2). Furthermore, there was a significant positive correlation between the phenolic content and flavonoid content of the extract and the *α*-amylase, *β*-glucosidase and pancreatic lipase inhibition, with (r = 0.97 *p* = 0.007 and r = 0.91 *p* = 0.008) for *α*-amylase inhibition, (r = 0.93 *p* = 0.006 and r = 0.90 *p* = 0.0073) for *β*-glucosidase inhibition and (r = 0.92 *p* = 0.005 and r = 0.91 *p* = 0.006) for pancreatic lipase inhibition, demonstrating that phenolics and flavonoids may be the main constituents of the inhibitory effect of AEEE.

In this investigation, an abnormal serum lipid profile was established in diabetic rats. This agrees with [48,49]. This abnormal serum lipid profile was inverted after the incorporation of AEEE at both doses. Hence, the extract could be helpful in refining lipid metabolism, which will, in turn, aid in protection against various diabetic complications.

The AEEE treatment of the diabetic groups significantly increased SOD activity and significantly inhibited the creation of TBARS as compared with the DC group. This may be attributed to its high phenolic and flavonoid content and is in agreement with the high in vitro antioxidant potentials of both the extract and new isolate according to the DPPH, ORAC and ferrous reducing assays. Furthermore, AEEE was considered safe because it decreased the levels of AST, ALT, creatinine and urea as compared to DC. The biochemical findings are in agreement with the histopathological modifications to the *β*-cells of the pancreas. Such histopathological modifications were decreased by the incorporation of AEEE extract at both doses. The current study agrees with previous studies on the antidiabetic effects of herbal extracts [50,51].

## 4. Materials and Methods

### 4.1. General

The following solvents were used for paper chromatography: (1) H_2_O; (2) 2% HOAc (acetic acid: H_2_O, 98:2); (3) BAW (*n*-BuOH–HOAc-H_2_O, 4:1:5, upper layer) and (4) BBPW (Benzene-*n*-BuOH–Pyridine– H_2_O, 1:5:3:3, upper layer). A Shimadzu UV–Visible-1601 spectrophotometer was used to measure UV. The HRESI mass spectra were recorded on a Finnigan LTQ FT Ultra mass spectrometer (Thermo Fisher Scientific, Bremen, Germany). An NMR spectroscopical analysis was performed on a Brucker 400 MHz NMR spectrometer at 400 MHz [52]. Regarding the materials, the chemicals, solvents, *α*-amylase, collagenase, *β*-glucosidase enzymes and reference drugs (orlistat and acarbose with purities > 95%) were obtained from Sigma-Aldrich (Merck, Darmstadt, Germany).

### 4.2. Plant Materials

The aerial parts of AE were collected during the flowering stage in September 2020, from a rice field in Benha district, Qalubia governorate, Egypt. The voucher specimen (3468) was deposited in the Herbarium of the Flora and Phytotaxonomy Research Department (CAIM), Horticultural Research Institute, Agricultural Research Center, Giza, Egypt. The specimen’s identity was verified by Prof. Dr. Abdel-Haleem Abdel-Mogaly, Prof. of Botany at the Agricultural Research Centre.

### 4.3. Preparation of AEEE

The aerial parts of AE (2.5 kg) were extracted via refluxing with EtOH/H_2_O (3:1, 3 times, each with 3 L, for 8 h). The solvent was removed under reduced pressure at 50 °C to yield a dark brown amorphous material (150 g).

### 4.4. Estimation of Total Phenolic and Flavonoid Contents

Folin–Ciocalteu reagent was used to measure phenolic content, which was estimated in gallic acid equivalents (GAE) per gram of sample. Aluminum chloride (AlCl_3_) was used to measure total flavonoid content (colorimetric assay), which was estimated in catechin equivalents (CE) per gram of sample [52].

### 4.5. Isolation and Identification of Phenolics (***1***–***5***)

A portion of AEEE (70 g) was applied to a Sephadex LH 20 column (480 g) and eluted with H_2_O/MeOH mixtures of decreasing polarity to yield seven major fractions (I–VII). Fraction I was eluted with H_2_O and II with 20%, III with 40%, IV with 50%, V with 70%, VI with 80% and VII with 90% MeOH. The collected fractions were individually subjected to two-dimensional paper chromatography (2DPC).

Compound **1** (77 mg) was isolated in pure form from fraction II (1.8 g) via repeated column fractionation over MCI gel (CHP-20P, 75–150 mm; Mitsubishi Chemical Co., Düsseldorf, Germany), using MeOH/H_2_O mixture (20%) for elution. Compounds **2** (88 mg) and **3** (64 mg) were individually separated from fraction IV (9.21 g) via MCI gel column fractionation, using H_2_O as solvent, followed by preparative paper chromatography (Prep. PC), using BAW as a solvent. Compound **4** (79 mg) was isolated in pure form from 1.1 g of fraction V by applying repeated Sephadex LH-20 column fractionation and elution with an H_2_O–EtOH (30:70) mixture. Compound **5** (89 mg) was obtained from fraction VII (3.35 g) through fractionation over a polyamide column and elution with a mixture of MeOH–C_6_H_6_–H_2_O (60:38:2).

#### Myricetin 3-*O*-β-^4^C_1_-(6”-*O*-Galloyl Glucopyranoside) 7-*O*-β-^4^C_1_ Glucopyranoside, MGGG, New Compound **1**

^1^H-NMR results for **1** were as follows: flavonol moiety: 6.3 (d, *J* = 2.5 Hz, H-6), 6.75 (d, *J* = 2.5 Hz, H-8), 7.5 (s, H2′ and H-6′), glucoside moiety at C-3: 5.35 (1H, d, *J* = 8.5 Hz, H-1″, anomeric glucose at flavonol C-3), 4.26 (l H, d, *J* = 12 Hz, H-6″a), 4.02 (l H, m, H-6″b); 3.22-3.9 (glucoside protons overlapped with hydroxyl and water protons), galloyl moiety: 7.02 (s, H-2′′′′ and H-6′′′′-galloyl protons), glucoside moiety at: C-7: 5.02 (H-1′′′, anomeric glucose at flavonol C-7). ^13^C NMR results were as follows: myricetin moiety: 156.9 (C-2), 134.4 (C-3), 177.6 (C-4), 1560.8 (C-5), 99.2 (C-6), 161.6 (C-7), 94.6 (C-8), 156. (C-9), 104.5 (C-10), 121.2 (C-1′), 109.7, (C-2′ and C-6′), 145.9 (C-3′ and C-5′), 138.9 (C-4′); 3-*O*-*β*-glucoside moiety: 102.9 (C-1′′), 74.4 (C-2′′), 76.5 (C-3′′), 69.8 (C-4′′), 76.6 (C-5′′), 63.3 (C-6′′); 7-*O*-*β*-glucoside moiety: 99.3 (C-1′′′), 73.2 (C-2′′′), 77.2 (C-3′′′), 69.5 (C-4′′′), 76.9 (C-5′′′), 60.16 (C-6′′′); galloyl moiety: 121.9 (C-1′′′′), 109.7 (C-2′′′′ and C-6′′′′), 145.5 (C-3′′′′and C-5′′′′), 1356.8 (C-4′′′′), 166.5 (C = O).

### 4.6. Molecular Modeling

The docking analysis was performed by using MOE 2019.0102 software [53] (Molecular Operating Environment (MOE), 2019.01; Chemical Computing Group ULC, 1010 Sherbooke St. West, Suite #910, Montreal, QC, Canada, H3A 2R7, 2021). The binding sites were generated from the co-crystallized ligands within the crystal protein obtained from the Protein Data Bank (https://www.rcsb.org/, accessed on 22 August 2021) (PDB codes: 2QV4-2ZOX-2OXE) [27,28,54]. To prepare the protein for the docking experiments, water molecules were removed. The crystallographic disorders and unfilled valence atoms were corrected using the protein report and the utility and clean protein modules. The protein geometry was corrected by applying the CHARMM and MMFF94 force fields. The rigidity of the binding site was obtained by applying a fixed atom constraint. The active site’s essential amino acids were defined and prepared for the docking process. The structures of the tested compounds (ligands) were imported in MDL-SD file format. The 3D structures of the ligands were prepared for docking, first by protonation and then via energy minimization by applying 0.05 RMSD kcal/mol using the CHARMM and MMFF94 force fields. The molecular docking experiment was carried out using the CDOCKER docking engine. The receptor was held rigid, while the ligands were allowed to be flexible. During the refinement stage, each molecule was allowed to produce ten poses with the protein. The docking scores (-CDOCKER interaction energy) for each docking experiment were recorded. The output from MOE 2019.0102 was further visualized with Discovery Studio 2019 software [55]. The molecular docking algorithm was initially validated via the redocking of the co-crystallized ligands into the active site of the respective receptors, with the calculation of root mean square deviation (RMSD) for the reliability and reproducibility of the proposed docking algorithm [56]. The crystal ligands of *α*-amylase, *β*-glucosidase and pancreatic lipase were redocked, and the RMSD value, with agreement within 2.0 Å indicating a validated algorithm, was compared to the tested compounds (**1**–**5**).

### 4.7. In Vitro Studies

#### 4.7.1. 2,2-Diphenyl-1-picrylhydrazyl (DPPH) Assay

The assay was carried out for AEEE and isolated phenolics according to Brand-Williams et al. [57].

#### 4.7.2. Oxygen Radical Absorbance Capacity (ORAC Assay)

The antioxidant assay was applied to AEEE and the isolated phenolics [58].

#### 4.7.3. Reducing Power Assay

The assay was carried out on AEEE and the isolated phenolics (DeGraft-Johnson et al., 2007).

#### 4.7.4. α-Amylase Inhibition

The assay was implemented in accordance with [50,59]. The positive control (acarbose) and tested samples were used in a concentration range of (75–600 µg/mL). The percentage of inhibition can be estimated using the following Equation:

100−[{A sample/ Acontrol ×100]

#### 4.7.5. β-Glucosidase Inhibition

The assay was performed in conformity with [60] and used the same formula for amylase. The positive control (acarbose) and tested samples were used in a concentration range of 75–600 µg/mL.

#### 4.7.6. Pancreatic Lipase Inhibition

The determination of the percentage inhibition of pancreatic lipase was calculated as prescribed by Hegazi et al. [50], using the same formula for amylase. The positive control (orlistat) and tested samples were used in a concentration range of 12–100 µg/mL.

### 4.8. In Vivo Studies

#### 4.8.1. Experimental Animals

Male Sprague-Dawley rats (170–220 g) were acquired from the National Research Centre (NRC, Giza, Egypt). The animals were acclimatized in our animal facility for one week before the experiment. The animals had total access to standard laboratory food pellets and water ad libitum under temperature-controlled conditions and 12 h light-dark cycles. The animal experiments were conducted according to the international regulations on the usage and welfare of laboratory animals and were approved by the Ethics Committee of the National Research Centre, Cairo, Egypt, Protocol number 49/261 (2019).

#### 4.8.2. Acute Oral Toxicity

The acute oral toxicity of AEEE was set for male Sprague-Dawley rats according to OECD guideline No.423 (OECD, 2001). Based on a pilot study in our laboratories, a limit test was performed. Animals fasted overnight, and the extract was administered orally using a gastric feeding needle at a dose of 2000 mg/kg (10 mL/kg dosing volume) [61].

#### 4.8.3. Induction of Diabetes

Type 2 diabetes was induced via two consecutive injections of nicotinamide (NA) and streptozotocin (STZ). The NA was dissolved in normal saline. Overnight fasted rats were injected intraperitoneally (i.p.) with NA (110 mg/kg) 15 min before an (i.p.) injection of a freshly prepared solution of STZ (25 mg/kg) in 0.1 M-citrate buffer (pH 4.5) (Reference 20459021). All rats were injected with STZ–NA, except the negative control rats, which received only the vehicle, distilled water. After 6 h of NA injection, the rats were provided with free access to glucose solution (10%, w/v) for the next 24 h. After 48 h of STZ administration, tail vein blood was collected to determine the rats’ fasting blood glucose levels calorimetrically (Diamond Diagnostics, Cairo, Egypt) [51]. Rats with glucose levels over 200 mg/dL were considered diabetic and included in the study.

#### 4.8.4. Experimental Design

Male Sprague-Dawley were randomly divided into six groups, with each group being comprised of six rats, as follows: Group I: normal control rats (NC); Group II: normal rats treated with AEEE (500 mg/kg) (AE 500-NC); Group III: diabetic control (DC); Group IV: diabetic rats treated with AEEE (250 mg/kg) (AE250-DC); Group V: diabetic rats treated with AEEE (500 mg/kg) (AE500-DC); Group VI: diabetic rats treated with the standard drug, glibenclamide (0.25 mg/kg). Groups I and III received only the vehicle (distilled water). The administration of different oral doses of AEEE began 72 h after STZ injection. This was performed using an intragastric tube for the treated group daily until the experiment ended. Weight measurement was performed at the beginning of the study and at the end of the 28th day. Doses were chosen based on the previous literature [37].

#### 4.8.5. Blood and Tissue Sampling

Fasting blood glucose FBG was measured 14 and 28 d after treatment. After the 28th day, blood samples were taken from the retro-orbital venous plexus under light ether anesthesia after overnight fasting. Pancreatic tissues were dissected. They were washed in ice-cold saline solution immediately. After that, they were divided into two portions. One was homogenized in 0.1 mol/L potassium phosphate buffer (pH 7.4) using Tissue Master TM125 (Omni International, Atlanta, GA, USA). After centrifugation at 3000 r/min for 10 min, the clear supernatant was kept at −80 °C for biochemical assays. The second portion was placed in 10% formalin for histopathological investigation [62].

#### 4.8.6. Assay of Biochemical Markers

##### Determination of Liver and Kidney Functions Markers

Serum aspartate transaminase (AST), alanine transaminase (ALT), urea and creatinine levels were measured as kidney function tests using the kits provided by the Spectrum Diagnostics Company (Egypt). The operational processes were measured in accordance with the kit instructions.

##### Determination of Insulin and α-Amylase Activity

Insulin level was determined using an ELISA kit (CUSABIO, Wuhan, China), and *α*-Amylase activity was assessed by ELitech Clinical Systems (Sèes, France).

##### Measurement of Serum Lipid Profile

Triacylglycerol (TAG), total cholesterol (TC) and high-density lipoprotein cholesterol (HDL-C) were assayed calorimetrically using Reactivos GPL (Barcelona, Spain). Low-density lipoprotein cholesterol (LDL-C) was calculated from TAG and HDL-C values according to Friedewald’s formula [63]:

LDL−C=(TC)−(HDL−C)−(TGs/5)

##### Determination of Oxidative Stress Markers in Pancreatic Tissue

Superoxide dismutase activity (SOD) was estimated in accordance with Minami and Yoshikawa [64]. Lipid peroxidation was measured using thiobarbituric acid reactive substances (TBARS) calorimetrically [65].

#### 4.8.7. Histopathological Investigation

The histopathologic examination was carried out on pancreas specimens (in 10% formalin) using light microscopy. Then, samples were processed to obtain 5 µm thick paraffin sections, followed by staining with hematoxylin and eosin (E) and observation under a Leica photomicroscope.

### 4.9. Statistical Analysis

Results are expressed as means ± standard deviationss (SDs). The differences between the various groups were analyzed using a one-way analysis of variance (ANOVA), followed by Tukey’s post-hoc test. The level of significance was set at *p*-values ≤ 0.05. All analyses were performed using the SPSS Ver. 25.0 (IBM, Chicago, IL, USA).

## 5. Conclusions

This is the first study to investigate the phenolic constituents of a flowering whole-plant ethanol extract of AE, in addition to the isolation of myricetin 3-*O*-*β*-*^4^C_1_*-(6′′-*O*-galloyl glucopyranoside)-7-*O*-*β*-*^4^C_1_*-glucopyranoside (MGGG), which has not been reported previously in nature. According to the results, AEEE effectively improved hyperglycemia and lipid profile while reducing oxidative stress in diabetic animals, with no signs of toxicity. In addition, histopathological investigations demonstrated a decrease in pancreas damage, which was aligned with the biochemical findings. This result obviously indicates the potential efficacy of AEEE in the management of T2D. In addition, this result suggests antioxidant activity, the inhibition of digestive enzyme actions and in vivo antidiabetic activity as potential pathways for the AE treatment of T2D. This encourages further investigations of AEEE to discover its mechanism of action at the molecular level and the signaling pathways involved, in addition to prospective prophylaxis against and/or curing diabetes.

## Figures and Tables

**Table 1:** Analysis results of isolated compounds (**1**–**5**) against (*human pancreatic α-amylase*) target site PDB ID: 2QV4.

Ligand	RMSD Value (Å)	Docking Score (kcal/mol)	Interactions and Residues	Distance (Å)
**Acarbose**	1.99	−8.131	Hydrogen bonding: Thr163 His305 His201 Asp300	2.42 2.67 1.97 2.37 1.83 1.97
			π- interactions: Leu165 His101	-
**Compound 1**	1.98	−8.99	Hydrogen bonding: Asp300 Asp197 π- interactions: His305 Trp59 Ile235 Lleu162	2.84 2.17 2.31 -
**Compound 2**	1.94	−7.07	Hydrogen bonding: His101 Asp197 Asp300 Gln63 Thr163	2.45 2.24 1.97 2.15 3.08 2.69
			π- interactions: Leu162	-
**Compound 3**	1.69	−6.43	Hydrogen bonding: His101 Asp197 Thr163 π- interactions: Leu162,Asp300,Val107	2.72 1.83 2.11 -
**Compound 4**	2.39	−6.47	Hydrogen bonding: His101 Asp300 Gln63	2.98 2.46 2.54 2.23 4.85
			π- interactions: Tyr151	-
**Compound 5**	1.88	−6.50	Hydrogen bonding: Tyr62 Gln63 Tyr151 Glu233 His305 Tyr163	2.01 3.06 2.85 2.52 2.33 1.83
			π- interactions: Leu165,Leu162.Ala198,Ile235	-

**Table 2:** Analysis results of isolated compounds (**1**–**5**) against (*human β-glucosidase*) target site PDB ID: 2ZOX.

Ligand	RMSD Value (Å)	Docking Score (kcal/mol)	Interactions and Residues	Distance (Å)
**Acarbose**	1.77	−5.04	Hydrogen bonding: Lys278 Asn319 Gln271 Ser270 Glu331	2.68 2.50 2.82 2.68 2.36
**Compound 1**	1.88	−6.43	Hydrogen bonding: Lys321 Glu318 Asn319 Met275 Gln279 Lys278 Gln271 π- interactions: Met275,Lys278	2.12 2.41 2.28 2.65 2.94 2.90 2.91 2.78 -
**Compound 2**	2.03	−5.28	Hydrogen bonding: Tyr316 Glu331 Asn319 Lys278	1.92 2.22 2.73 2.10 2.46 2.74 2.02
**Compound 3**	2.56	−5.42	Hydrogen bonding: Glu318 Lys278 Gln271 Tyr316 Glu241 Arg245	2.10 2.50 2.04 5.02 1.93 2.24 3.07 3.07
**Compound 4**	1.68	−5.36	Hydrogen bonding: Lys320 Lys278	5.26 3.34 2.81
			π- interactions: Tyr316	-
**Compound 5**	1.18	−5.70	Hydrogen bonding: Glu318 Ser238 Lys315 Gln271 Lys278	1.96 1.98 2.41 2.78 2.22 2.85 2.29
			π- interactions: Glu331	-

**Table 3:** Analysis results of isolated compounds (**1**–**5**) against (*human pancreatic lipase*) target site PDB ID: 2OXE.

Ligand	RMSD Value (Å)	Docking Score (kcal/mol)	Interactions and Residues	Distance (Å)
**Orlistat**	1.24	−6.66	Hydrogen bonding: Gln388 Asn385	2.85 2.26
			π- interactions: Leu463 Lys419	-
**Compound 1**	1.78	−7.70	Hydrogen bonding: Asn353 Glu350 Thr355 Asp409 Asn295 π- interactions: Asp409	1.96 1.83 2.13 2.79 2.61 -
**Compound 2**	1.99	−7.20	Hydrogen bonding: Glu350 Ser351 Asp409 Thr355	2.88 2.02 2.88 2.20 2.49
**Compound 3**	1.60	−7.39	Hydrogen bonding: Asn385 Thr355 Asp409 Glu350 Asn353 Asn295 Asn411 Asp297 π- interactions: Phe410	3.10 2.38 2.07 2.06 2.42 2.14 2.38 2.87 -
**Compound 4**	2.16	−5.43	Hydrogen bonding: Asn411 Gln384 Thr355 Asp409 Ser351 Glu350	2.34 2.88 2.08 2.09 2.15 2.98 2.03
**Compound 5**	2.03	−5.51	-	3.02 2.01 1.92 2.5

**Table 4:** Effect of different tested compounds in the three different enzyme inhibition assays.

*α*-Amylase Inhibition at 300 µg/mL			
**Tested Sample**	**% Inhibition**	**IC_50_ µg/mL**	***p* Value**
AEEE	89.02 ± 2.31	176.3 ± 4.21	
MGGG	91.36 ± 2.45	157.54 ± 5.9	<0.0001
Kaempferol 3-*O*-rutinoside	80.37 ± 3.81 *	206.89 ± 5.60	<0.0001
Quercetin 3-*O*-rutinoside	72.13 ± 3.49	223.6 ± 3.90	<0.0001
Tellimagranidine-I	78.92 ± 4.13	217.35 ± 5.30	<0.0001
2,3-*α*, *β*-digalloy glucose	78.04 ± 1.15	216.89 ± 5.50	<0.0001
Acarbose 300 ug/mL	93 ± 1.80	71.85 ± 3.70	<0.0001
*β*-glucosidase inhibition at 300 µg/mL			
AEEE	88.13 ± 1.25	76.12 ± 2.25	<0.0001
MGGG	92.70 ± 1.42 *	72.77 ± 2.67	<0.0001
Kaempferol 3-*O*-rutinoside	47 ± 4.79 *	140.33 ± 5.20	<0.0001
Quercetin 3-*O*-rutinoside	66 ± 3.47 *	104 ± 3.80	<0.0001
Tellimagranidine-I	58.44 ± 3.61 *	115.21± 4.90	<0.0001
2,3-*α*, *β*-digalloy glucose	68.43 ± 2.30 *	100.23 ± 3.80	<0.0001
Acarbose at 600 µg/mL	95.2 ± 2.30	110.6 ± 3.20	<0.0001
Pancreatic Lipase Inhibition at 100 µg/mL			
AEEE	79 ±3.21	45 ±5.11	
MGGG	87 ± 2.38 *	32 ± 4.43 *	<0.0001
Kaempferol 3-*O*-rutinoside	35.81 ± 3.84 *	135.8 ± 4.30 *	<0.0001
Quercetin 3-*O*-rutinoside	82.17 ± 2.53 *	40.42 ± 2.41 *	<0.0001
Tellimagranidine-I	19.52 ± 4.36 *	270.1 ± 6.20 *	<0.0001
2,3-*α*,*β*-digalloy glucose	18.69 ± 3.10 *	265.95± 4.71 *	<0.0001
Orlistat 100 ug/mL	57 ± 4.50	3.22 ± 1.30	<0.0001

Data are presented as % of inhibition ± S.D. and IC_50_ ± S.D; *: indicates significant difference in % of inhibition between AEEE and its isolated compounds.

## Data Availability

The data presented in this study are available in the article and Supplementary Material.

## Supplementary Materials

The supporting information can be downloaded at: https://www.mdpi.com/article/10.3390/plants11030452/s1.
