# Supplementary figures and images for: Antidiabetic Activity and In Silico Molecular Docking of Polyphenols from Ammannia baccifera L. subsp. Aegyptiaca (Willd.) Koehne Waste: Structure Elucidation of Undescribed Acylated Flavonol Diglucoside
Source: Plants (Basel). 2022 Feb 6;11(3):452. doi: 10.3390/plants11030452 (PMC8840488; doi:10.3390/plants11030452)

$^1\text{H}$  NMR of Compound (1)

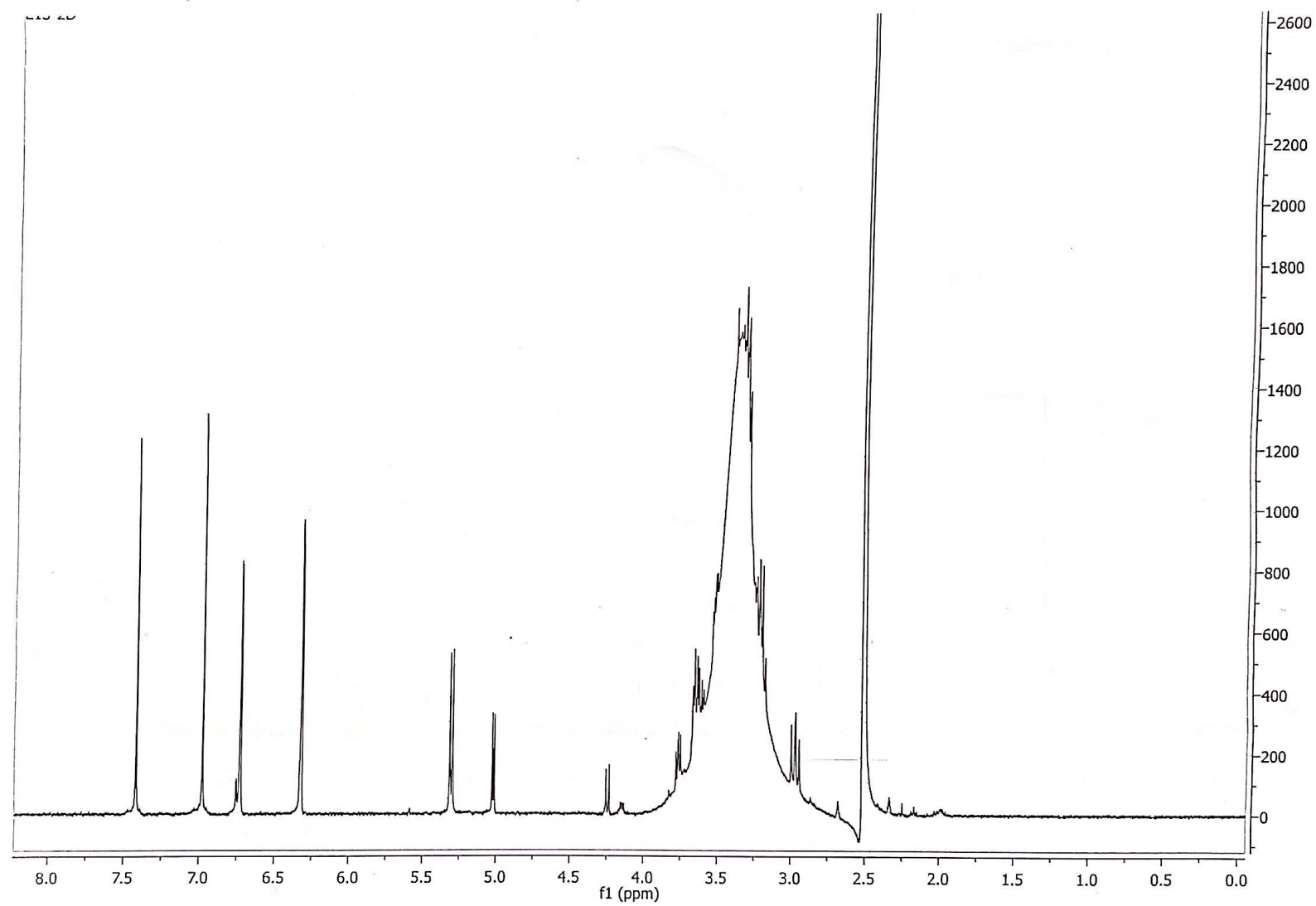

$^{13}\text{C}$  NMR of Compound (1)

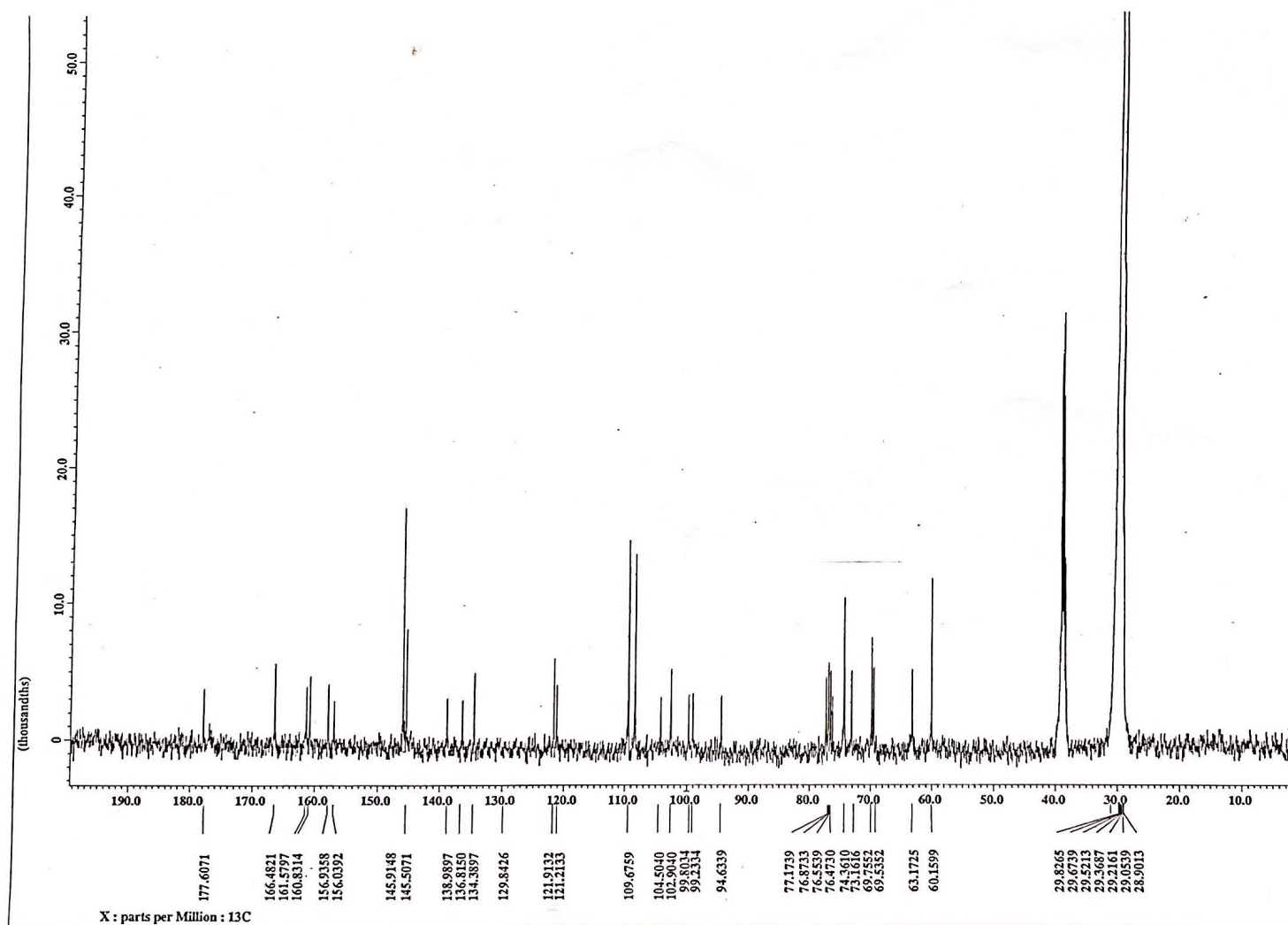

HMBC of Compound (1)

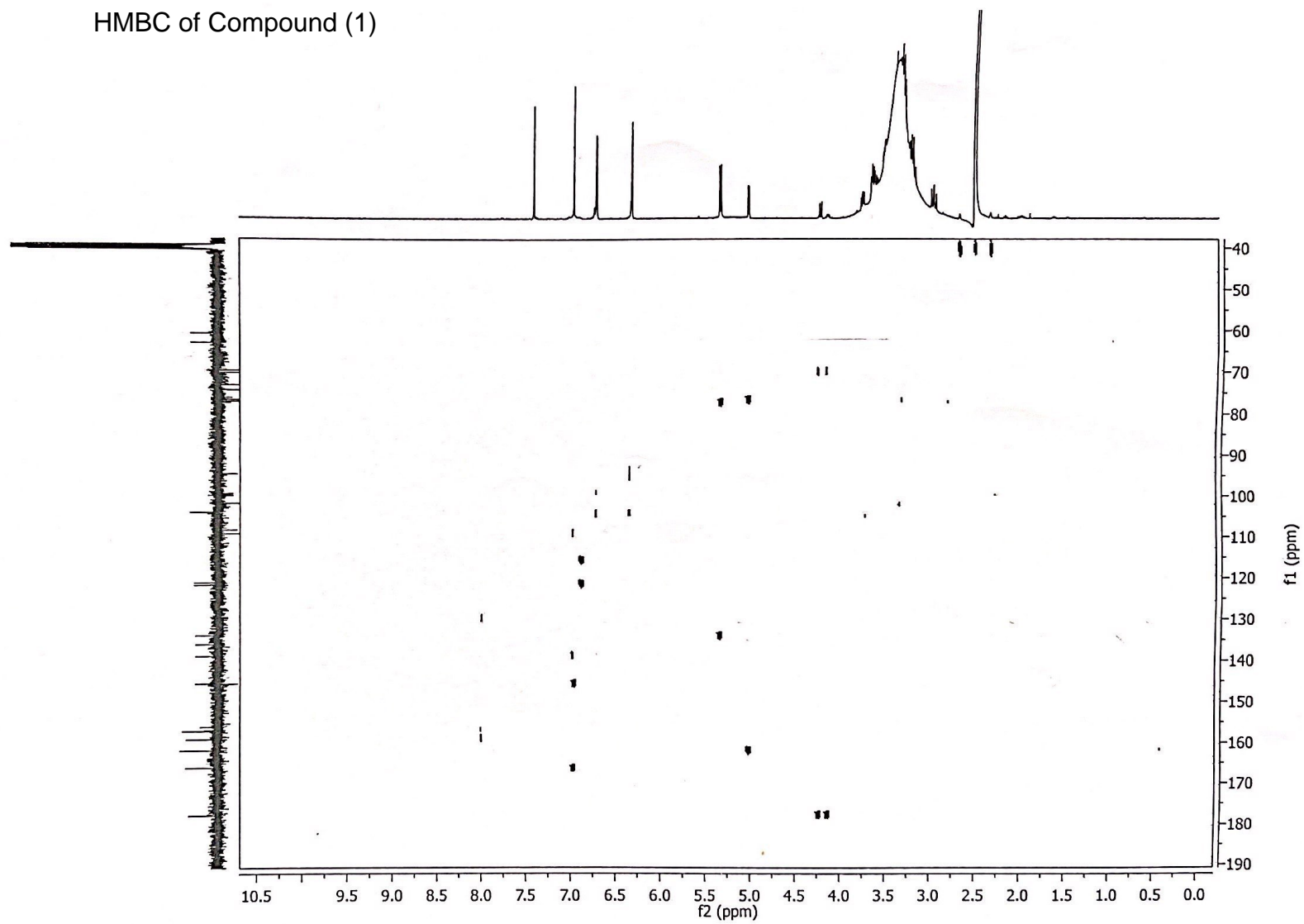

HSQC of Compound (1)

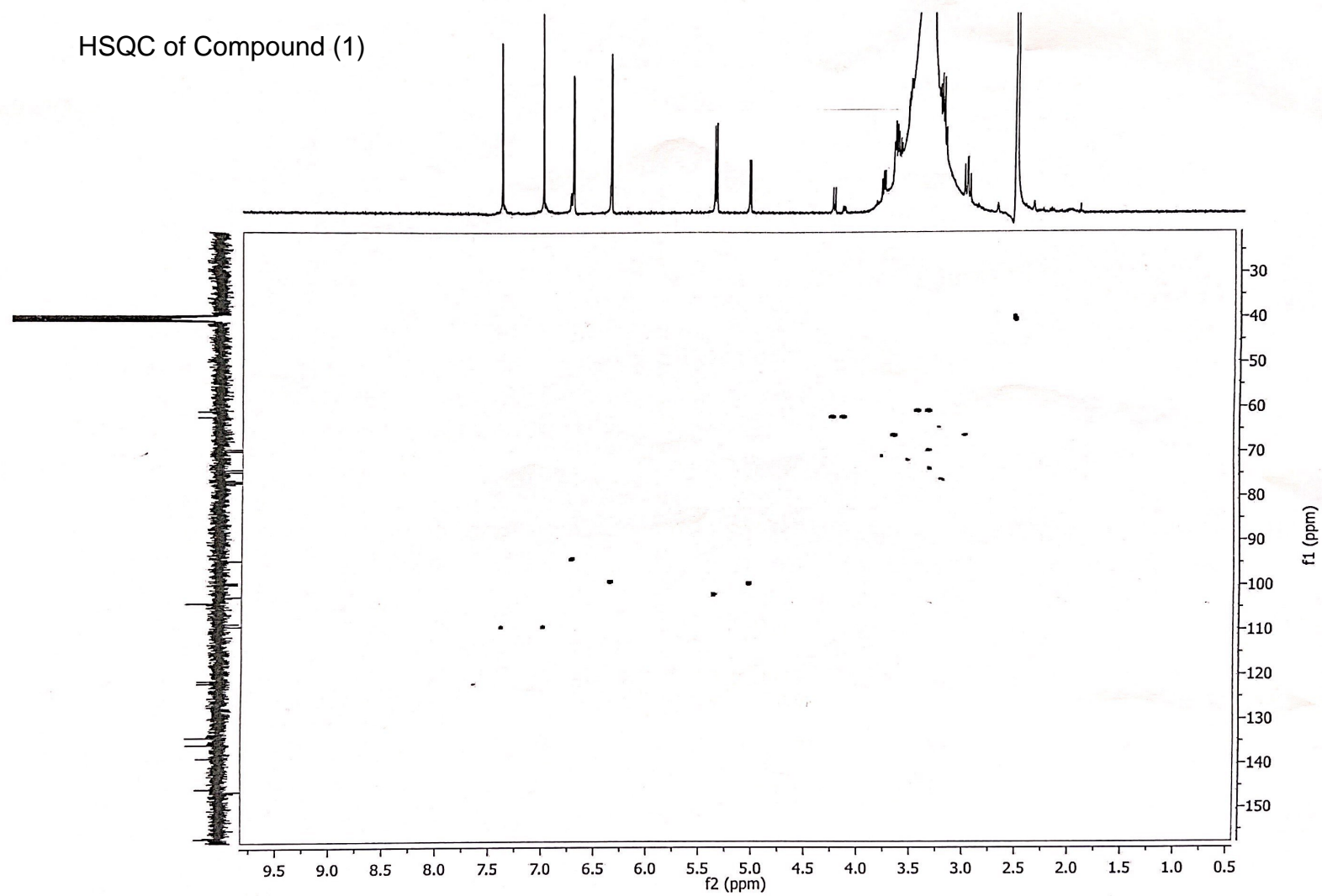

# ESI MS of Compound (1)

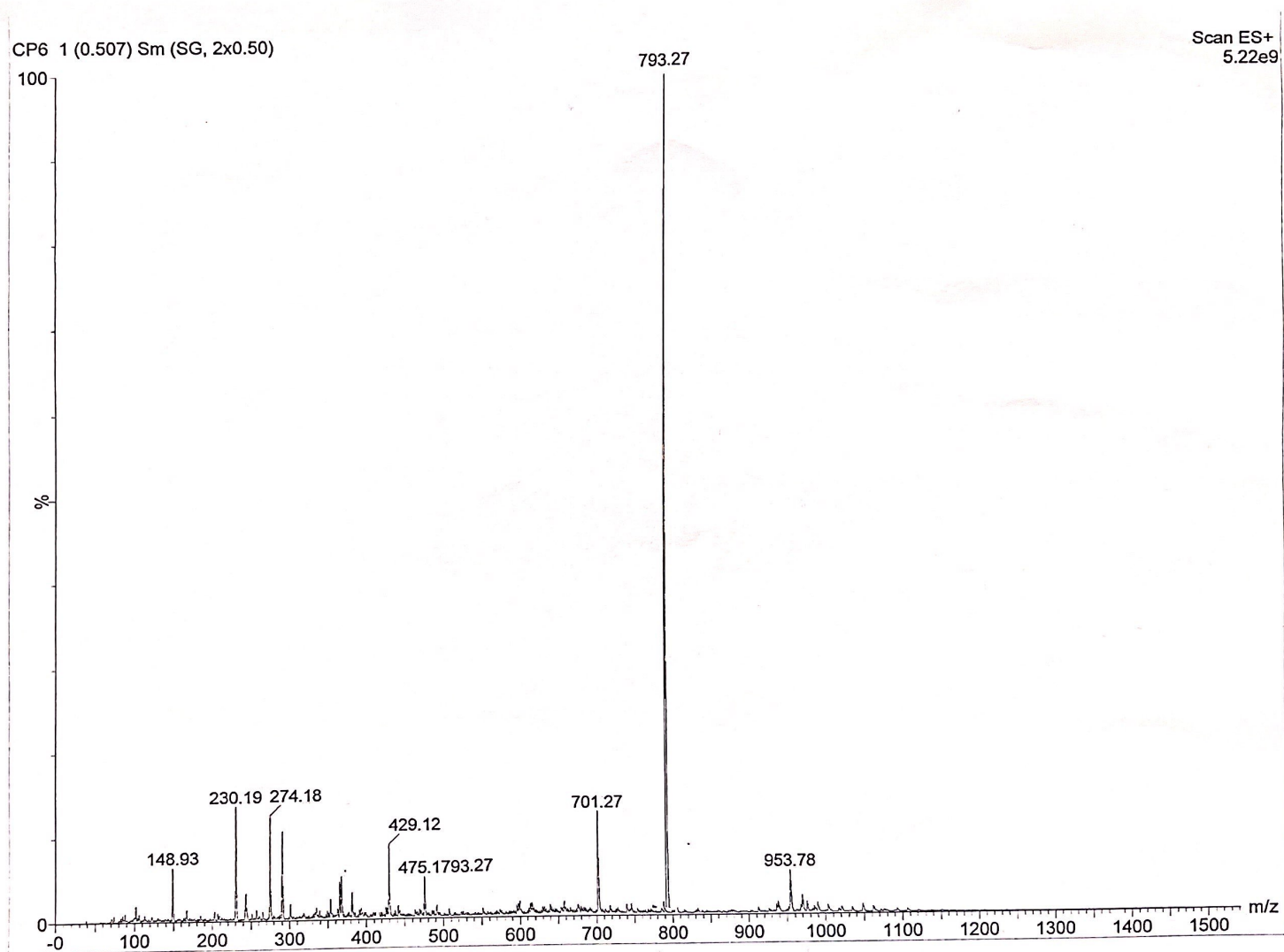

Supplement: Supplementary file 1 [file plants-11-00452-s001.zip › plants-1533791-supplementary.pdf]
